# Two sides of the same coin? Patient and therapist experiences with a transdiagnostic blended intervention focusing on emotion regulation

**DOI:** 10.1016/j.invent.2022.100586

**Published:** 2022-11-10

**Authors:** Laura Luisa Bielinski, Oliver Thomas Bur, Gwendolyn Wälchli, Jeannine Michelle Suter, Nathalie Walsh, Marijke Amanda Kley, Tobias Krieger, Thomas Berger

**Affiliations:** Department of Clinical Psychology and Psychotherapy, University of Bern, Fabrikstrasse 8, 3012 Bern, Switzerland

**Keywords:** Blended therapy, Transdiagnostic, Emotion regulation

## Abstract

**Introduction:**

The combination of internet-based intervention and psychotherapy, commonly termed blended therapy (BT), has gained popularity in recent years. While advantages and disadvantages of BT have been identified from the patient and therapist perspective, the two perspectives have rarely been examined within the same treatment. Moreover, almost all available research on patient and therapist experiences with BT is disorder-specific. This study aimed to investigate patient and therapist experiences within the same transdiagnostic BT.

**Methods:**

A qualitative analysis of semi-structured interviews with eight patients and eight therapists taking part in a transdiagnostic blended intervention focusing on the topic of emotion regulation was conducted. A qualitative content analysis approach was used. Category frequencies were calculated and similarities and differences between the patient and therapist experience were explored.

**Results:**

Ten main themes and 59 subthemes were identified in the category system for patient interviews and ten main themes and 50 subthemes were identified in the category system for therapist interviews. Similarities and differences between the two perspectives were reported with regard to 1) expectations toward the intervention, 2) the internet-based intervention, 3) symptomatology and emotion regulation, 4) the therapeutic relationship and 5) the blended format.

**Conclusion:**

This study provides first insights on the experiences with transdiagnostic BT focusing on emotion regulation. Based on the results, different recommendations for the improvement of transdiagnostic BT are made. Future research on patient and therapist experiences with transdiagnostic BT is necessary, in order to further improve the experience of those involved.

## Highlights

• First paper to describe both patient and therapist experiences with transdiagnostic BT focusing on emotion regulation in an outpatient psychotherapy setting
• Similarities and differences between the patient and therapist experience are explored
• Recommendations for the improvement of patient & therapist experience with transdiagnostic BT are made

## 1 Introduction

### 1.1 Background

In the last two decades, studies have provided evidence for the efficacy and effectiveness of the combination of face-to-face (FTF) psychotherapy and internet-based interventions (IBI), commonly termed *blended therapy (BT)* (Berger et al., 2018; Thase et al., 2018). BT can be realized in different ways ranging from IBIs provided as stepped care (Nordgreen et al., 2016), aftercare (Hennemann et al., 2018) or add-on (Berger et al., 2018) to more intricate combinations of psychotherapy and internet-based content (Nakao et al., 2018).

Depending on the nature of the blend, different benefits of BT relative to other forms of treatment delivery exist. Compared to FTF therapy, these may include more effective treatment (Berger et al., 2018), a saving of therapist time (Thase et al., 2018), and potentially also improved cost-effectiveness (Wright et al., 2005). Compared to stand-alone IBIs, these may include better suitability in acute crises, or greater acceptability amongst stakeholders (Topooco et al., 2017).

### 1.2 Patient and therapist experiences with disorder-specific BT

Stakeholder experiences with disorder-specific BT have been examined in several studies. van der Vaart et al. (2014) collected survey data from therapists and patients from mental health institutions in the Netherlands. The largest percentage of patients agreed with the following benefits of BT: the convenience of access to therapy content, BT encouraging patients to take more responsibility for (succeeding of) therapy, and the ability to see a patient/therapist FTF. The largest percentage of therapists agreed with the following benefits of BT: the convenience of access to therapy content, therapy blending into the patients' home/private situation, and sessions being completed in patients' own time. The drawbacks that the largest percentage of both therapists and patients agreed with were that BT is not suitable for every patient and could cause interpretation problems due to the lack of non-verbal communication. Importantly, part of the study sample had no experience with BT.

Concerning actual experiences with BT, studies have reported on the patient perspective. In a sample of 15 depressed patients who had been treated with a blended internet and video-based protocol in routine care (Etzelmueller et al., 2018), semi-structured interviews were conducted. Patients were generally satisfied with the treatment, described it as useful, and highlighted the individualized nature of treatment along with the guidance component as particularly positive. Some of the disadvantages mentioned were technical difficulties and a sense of distance created by the video format.

Urech et al. (2019) used a qualitative content analysis (Mayring, 2015) to analyze patient interviews. Depressed individuals were provided with a combination of internet-based modules and FTF sessions. Patients' perceived advantages included the complementary nature of the format, the therapist support for the online components, the ability to outsource specific treatment components to the internet-based parts, and the efficient nature of the treatment compared to FTF therapy. Disadvantages included low motivation to work on the online program, the lack of individualization of the program's content, and a lack of interplay between FTF sessions and IBI (Urech et al., 2019).

Benefits and drawbacks have also been reported from the therapist's perspective. Interviews with five therapists providing BT for depression were examined via qualitative content analysis (Titzler et al., 2018). Twenty-nine barriers and 33 facilitators for implementing BT were identified. Facilitators mentioned by all therapists regarding patient factors were patient interest and motivation to participate. Facilitators mentioned by all therapists regarding therapist and therapeutic factors included amongst others: time savings in therapy, access to internet-based programs between FTF sessions and the structure of the internet-based program guiding the treatment. Barriers regarding therapist or therapeutic factors mentioned by all therapists included therapeutic alliance burdened by technical issues, limited number of FTF sessions hindering the therapy process, limited customizability and autonomy in decisions, negative affect caused through technical difficulties, and negative effects and time burden. Barriers regarding patient factors included reservations and less engagement in internet-based programs and disease-related contraindication (Titzler et al., 2018).

A comprehensive investigation of therapists with different experience levels providing BT for depression, revealed that therapists were generally satisfied with providing BT. The most cited positive experience was the containing of therapist drift due to the preset structure. Negative aspects included higher costs, unsuitability of content, and no time gains for therapy (Mol et al., 2020).

In summary, patient and therapist experiences with BT include positive (e.g., accessibility of content, complementary nature of the format) and negative aspects (e.g., difficulties with technology, questions about indication), with the overall response being relatively positive. However, while the aforementioned studies all focus on the disorder-specific treatment, little is known about experiences with transdiagnostic BT.

### 1.3 Transdiagnostic treatment

Several definitions of transdiagnostic treatment are available in the literature (Mansell et al., 2009; McEvoy et al., 2009; Sauer-Zavala et al., 2017). Definitions have in common that transdiagnostic treatments are provided across different disorders or diagnoses and do not require diagnosis-specific information to be effective (McEvoy et al., 2009; Mansell et al., 2009). Several of these types of treatments also target mechanisms or processes independent of diagnoses, commonly termed transdiagnostic processes (Schaeuffele et al., 2021). Such processes are present across various disorders, causally contributing to the development or maintenance of psychopathology (Harvey et al., 2004). Examples of transdiagnostic processes may include but are not limited to several cognitive and behavioral processes (Harvey et al., 2004), perfectionism (Egan et al., 2011), psychological inflexibility (Levin et al., 2014) and emotion regulation (Berking and Wupperman, 2012).

### 1.4 Emotion regulation

Emotion regulation is one example of a transdiagnostic process that may be relevant to the development, maintenance, and treatment of different forms of psychopathology (Berking and Wupperman, 2012). Emotion regulation can be defined as the processes by which we influence which emotions we have, when we have them, and how we experience and express them (Gross, 1998). According to the extended process model (EPM), the emotion regulation process can be divided into the identification, selection, and implementation phases. The identification phase focuses on identifying whether to regulate an emotion. The selection phase focuses on the selection of an emotion regulation strategy. Finally, the implementation phase focuses on translating strategies into specific tactics. The EPM has also been used to guide the development of clinical interventions (Gross, 2015).

### 1.5 Transdiagnostic BT focusing on emotion regulation

BT offers one possible way of incorporating an intervention focusing on emotion regulation into psychotherapy. Interestingly, preliminary data from a study investigating transdiagnostic blended CBT for emotional disorders in a group setting, suggests acceptance of the intervention amongst individuals with anxiety and depressive disorders (Díaz-García et al., 2020). BT focusing on emotion regulation in the individual psychotherapy setting is currently being examined in a pilot RCT (Bielinski et al., 2020). This type of intervention may benefit patients by allowing them to work on emotion regulation content independent of place and time, in addition to their FTF sessions. The combination may also help therapists by freeing up more time in face-to-face sessions for process-related content (van der Vaart et al., 2014) or for disorder-specific content. On the other hand, negative effects may also be experienced by both patients and therapists. These could range from problems with technology (Etzelmueller et al., 2018; Titzler et al., 2018) to issues that are more specific to the transdiagnostic nature of the IBI, such as content not being relevant to a patients' specific problem.

### 1.6 The current study

By analyzing interview data from a pilot RCT (Bielinski et al., 2020), this paper aims to shed light on positive and negative experiences reported by patients and therapists following transdiagnostic BT. To the best of our knowledge, no previous study has examined both patient and therapist experiences with BT focusing on emotion regulation in an individual outpatient setting. Based on the results, recommendations for improving the patient and therapist experience with BT are made.

## 2 Method

### 2.1 Trial and participant recruitment

The current qualitative analysis is part of a pilot RCT investigating a blended transdiagnostic intervention for symptom reduction and improvement of emotion regulation in an outpatient psychotherapeutic setting (Bielinski et al., 2020). The main aim of the pilot RCT is to investigate a new blended approach with regard to feasibility and preliminary effects. In the trial, individuals treated at the outpatient clinic of the University of Bern are randomized to either 1) treatment as usual (TAU), which consists of integrative cognitive behavioral therapy at the outpatient clinic (Grawe, 2004) or 2) the intervention group that receives TAU plus access to an IBI focusing on emotion regulation. Patients are recruited via the outpatient clinic from February 2020 to randomize 35 individuals into the intervention group and 35 individuals into TAU. The trial was approved by the ethics committee of the Canton of Bern, Switzerland and registered in the Clinical Trials Registry, ClinicalTrials.gov NCT04262726.

Seventeen patients who had been consecutively randomized into the REMOTION arm of the trial and reached post-assessment (T1; 6 weeks) starting in May 2020 to December 2020, and their therapists, were asked about interview participation per e-mail. There was no compensation for taking part in the interview. Seven female and one male patient, aged 23 to 36 (*M* = 28.38, *SD* = 4.78) with a range of different diagnoses and six female and two male therapists, aged 28 to 35 (*M* = 31.75, *SD* = 3.01), agreed to provide information. A detailed description of the patient and therapist samples can be found in the Supplementary material. Interviewed patients completed significantly more IBI modules than non-interviewed patients. Importantly, there were no significant differences between the two samples concerning further key variables, including number of individuals with reliable symptom change on the BSI-GSI (Franke, 2000) at T1 (Supplementary material A).

### 2.2 Treatment

TAU consisted of integrative cognitive-behavioral therapy based on general psychotherapy principles (Grawe, 2004) as provided at the outpatient clinic of the University of Bern. The blended approach combined the internet-based intervention REMOTION (Bielinski et al., 2020) with FTF-TAU. REMOTION consists of six modules on an online platform, that follow the structure of the EPM (Gross, 2015). A detailed description of REMOTION can be found in a previous publication (Bielinski et al., 2020). Individuals are asked to complete one module per week, with open access to all modules from the beginning. Once a week, the study team sends an email to remind participants to work on the program. Therapists received information about the program's content in the form of an extended information booklet, including suggestions on how to integrate REMOTION content into FTF sessions. All therapies were conducted either by licensed psychotherapists or supervised psychotherapists in training.

### 2.3 Semi-structured interview and data collection procedure

Authors NW and JS, two master's degree psychology students, developed two semi-structured interview guides for this study (see Supplementary material B). All interviews except for one were conducted by telephone between May 2020 and April 2021 by authors JS and NW (from their home offices, audio recording) and transcribed using guidelines by Dresing and Pehl (2018). Before conducting the interviews, the two authors pilot tested the interview with first author LLB (doctoral student). At the beginning of each interview, NW and JS introduced themselves and the study goals, prompts were provided after each question if necessary. The duration of the patient interviews ranged from 16.01 min to 27.15 min (*M* = 19.67 *SD* = 4.42), the duration of therapist interviews ranged from 12.54 to 26.49 min (*M* = 16.28 *SD* = 4.93). One patient decided to provide written feedback instead of an audio interview. Anonymity was assured by using code numbers instead of names in the transcripts. Both JS and NW took part in an interview training session in-house before conducting the interviews. Both interviewers were familiar with the study protocol but blinded to interviewee questionnaire data at the time of the interview.

### 2.4 Data analysis

Interviews were analyzed with qualitative content analysis (Mayring, 2015), chosen for its systematic and rule-based approach, ease of integration with quantitative analysis elements and for its application in previous studies on BT also mentioned in the introduction section of this paper (Titzler et al., 2018; Urech et al., 2019). Using the inductive approach, codes were first developed from the transcripts guided by the research question: *Which experiences with blended intervention do patients/therapists report*? The resulting subthemes were then grouped into main themes using a deductive approach by considering previous qualitative research on experiences with BT (Urech et al., 2019; Mol et al., 2020) and theoretical constructs specific to the study (for example, emotion regulation, Gross, 2015). The smallest unit that could belong to one category was a one-word statement. A sentence or paragraph could be coded as containing aspects from one or more categories. We conducted separate qualitative analyses for patient and therapist interviews. The analyses followed the same methodological steps:

In a first step, four interviews (50 % of the material) were coded (JS coded four patient interviews, NW coded four therapist interviews). Initial codings were discussed and adapted accordingly (for example, different categories were rephrased, categories subsumed, or definitions of categories were amended and regrouped) with first author LLB. In a second step, all eight interviews were coded by the first author LLB, codings were again discussed with authors JS and NW, and the category system was again revised and adapted, as in the first step. In a third step, the general coding system based on all interviews (subthemes and main themes) was discussed with the last author TB (PhD, experienced psychotherapist and BT expert) and adapted again (slight rephrasing of certain categories for better understanding) until final consensus was reached.

The final category system for the patient interviews contained 59 subthemes and 10 main themes. The final one for the therapist interviews contained 50 subthemes and 10 main themes. In a final step, all interviews were then coded again with the final category system by first author LLB and by independent coders (GW for patient interviews and MK for therapist interviews). Inter-rater agreement was assessed using a Kappa formula suggested by Brennan and Prediger (1981, denoted κ*n*) and calculated with MAXQDA (VERBI, 2018). The inter-rater agreement analysis yielded an overall Kappa of κ*n* = 0.76 for the patient category system and κ*n* = 0.83 for the therapist category system. Finally, for each subtheme, the number of patients (or therapists) who mentioned a theme and the frequency of segments coded for that theme was analyzed based on first author LLB's codings (Supplementary material C).

In a further step, subthemes were labeled according to valence where applicable, and the similarities and differences regarding the subthemes mentioned by patients and therapists were analyzed. All interviews were transcribed and coded with MAXQDA 2018 (VERBI, 2018). All other quantitative analyses were conducted with SPSS version 27. The COREQ checklist (Tong et al., 2007) was used to ensure methodological quality.

## 3 Results

The complete category systems for patient and therapist interviews with definitions and illustrative quotations can be found in Supplementary material C. 3.1, 3.2 give an overview of the categories mentioned by patients and therapists. Section 3.3 provides an overview of similarities and differences between the patient and therapist experiences.

### 3.1 Patient experiences

Patient experiences are displayed in Table 1.

**Table 1:** Patient experiences (category system).

Main theme Subtheme	*N* patients (%)
Expectations/reasons for participation	
• No specific motivation or expectation	5 (62.5)
• Participation to support research	4 (50)
• Participation due to relevance of content/expectations toward content	3 (37.5)
• Participation due to expectations toward format	2 (25)
• Curiosity	2 (25)
• Participation to benefit from FTF sessions more	2 (25)
• Worry about independent work	1 (12.5)
• Participation due to pandemic	1 (12.5)
• Participation due to trust in therapist	1 (12.5)
IBI: User-friendliness and design	
• Aspects not user-friendly	7 (87.5)
• User-friendly	7 (87.5)
• Desire for a different format	5 (62.5)
• Too much information/effort	4 (50)
• Appealing design	3 (37.5)
• Not used enough	3 (37.5)
• Good amount of information	2 (25)
IBI: Content	
• Content & structure of content interesting	7 (87.5)
• Content too theoretical	2 (25)
• Theoretical background/connection to research positive	2 (25)
• Transdiagnostic nature positive	1 (12.5)
• Content not relevant to specific problem	1 (12.5)
IBI: Interactivity	
• Multifaceted design	4 (50)
• Videos and audio files positive	4 (50)
• Reminders helpful	3 (37.5)
• Characters positive	3 (37.5)
• Desire for more reminders and or guidance	3 (37.5)
• Reminders not helpful	2 (25)
• Characters negative	1 (12.5)
• Summaries positive	1 (12.5)
IBI: Exercises and practical application	
• Exercise content not helpful/too difficult	4 (50)
• Exercises too short or too little	4 (50)
• Transfer into daily life difficult	3 (37.5)
• Transfer into daily life successful	2 (25)
• Exercises helpful	1 (12.5)
• Practical application of content positive	1 (12.5)
Emotion & emotion regulation	
• More awareness toward own emotions	4 (50)
• Able to better influence own emotions	4 (50)
• Knowledge gain about emotions & emotion regulation	4 (50)
• No influence on emotion regulation	2 (25)
• Able to understand own emotions more	2 (25)
• Communication about emotions easier	1 (12.5)
Social environment	
• No change noticed by social environment	5 (62.5)
• Positive change noticed by social environment	3 (37.5)
• Positive influence on social interactions	1 (12.5)
Symptomatology	
• Positive influence on symptoms	4 (50)
• No symptom change observed	3 (37.5)
• Too early to tell if there is symptom change	2 (25)
Therapeutic relationship	
• No influence on therapeutic relationship	6 (75)
• Therapist has too little knowledge of intervention	3 (37.5)
• Better communication with therapist	1 (12.5)
• Independence from therapist strengthened	1 (12.5)
• Patient knowledge from IBI is problematic for therapist	1 (12.5)
Blended format	
• IBI and FTF not integrated enough	7 (87.5)
• IBI and FTF transform each other	6 (75)
• IBI and FTF complement each other	4 (50)
• IBI and FTF impede each other	3 (37.5)
• IBI does not replace FTF	2 (25)
• Wrong timing of intervention	2 (25)
• Patients see themselves as responsible for the integration of IBI and FTF	2 (25)

### 3.2 Therapist experiences

Therapist experiences are displayed in Table 2.

**Table 2:** Therapist experiences (category system).

Main theme Subtheme	*N* therapists (%)
Expectations/reasons for participation	
• Useful for patients in daily life	4 (50)
• No specific expectations	3 (37.5)
• Positive expectations toward format: Complements FTF	3 (37.5)
• Positive expectations toward format: Being able to outsource elements of FTF	2 (25)
• Patients learn skills	2 (25)
Previous experiences with BT	
• No previous experience	6 (75)
• Disorder specific experiences	2 (25)
IBI: Exercises	
• Exercises not helpful	2 (25)
• Exercises positive/helpful	1 (12.5)
IBI: Content and structure	
• Structure positive	5 (62.5)
• Thematic focus positive	4 (50)
• No changes needed	4 (50)
• Tempo and duration not suitable	2 (25)
• Not enough support in the IBI	2 (25)
• Too little content on emodiversity	1 (12.5)
• Too little content on connection between emotion, cognition, and behavior	1 (12.5)
IBI: Appealing design	3 (37.5)
Emotion & emotion regulation	
• Patients gain awareness toward emotions & emotion regulation	3 (37.5)
• Patients learn new strategies for emotion regulation/better influence own emotions	3 (37.5)
• Emotion regulation needs more time	3 (37.5)
• No influence on emotion regulation	2 (25)
• Patients show increased acceptance of emotions	1 (12.5)
Therapist information	
• Therapist material: Good content	6 (75)
• Therapist material: Appealing design and structure	5 (62.5)
• Therapist material: Too little information	5 (62.5)
• Personal access to IBI needed	4 (50)
• Information about patient progress in IBI needed	4 (50)
• Therapist material: Was not used or used too little	2 (25)
• Therapist material: Too much information	2 (25)
• Therapist material: Nothing missing	1 (12.5)
Symptomatology	
• Positive influence on symptoms	3 (37.5)
• Too early to tell if there is symptom change	2 (25)
• No symptom changes observed	1 (12.5)
Therapeutic relationship	
• No influence on therapeutic relationship	5 (62.5)
• Relationship strengthened due to broader therapy offer	3 (37.5)
• Negative influence due to increased workload for patients	2 (25)
• Negative influence because only available for patients in the outpatient clinic	1 (12.5)
• Less time needed for building of therapeutic relationship	1 (12.5)
• More relationship credit available for interventions in session	1 (12.5)
• Positive influence on therapeutic relationship since patients have more resources	1 (12.5)
• More contact with patients	1 (12.5)
Blended format	
• IBI and FTF not integrated enough	7 (87.5)
• IBI transforms FTF	7 (87.5)
- IBI enables new elements in FTF sessions	3 (37.5)
- IBI allows outsourcing of elements from FTF sessions	4 (50)
- IBI allows certain topics to be discussed earlier in FTF sessions	3 (37.5)
• No influence on session structure	6 (75)
• Patients integrated intervention into FTF	6 (75)
• Enables work independent of time and place for patient/useful in daily life	6 (75)
• Therapists integrated intervention into FTF	4 (50)
• Generally a positive experience	4 (50)
• IBI and FTF complement each other	4 (50)
• Flexible timing of intervention desired	2 (25)
• IBI does not replace FTF	1 (12.5)

### 3.3 Similarities and differences between the patients' and therapists' experiences

The following paragraphs highlight similarities and differences regarding expectations toward BT, the internet-based program, emotion regulation & symptomatology, the therapeutic relationship, and the blended format. Subthemes are divided into positive experiences (+) and negative experiences (−) where applicable.

#### 3.3.1 Expectations toward BT and reasons for participation

**Fig. 1:**
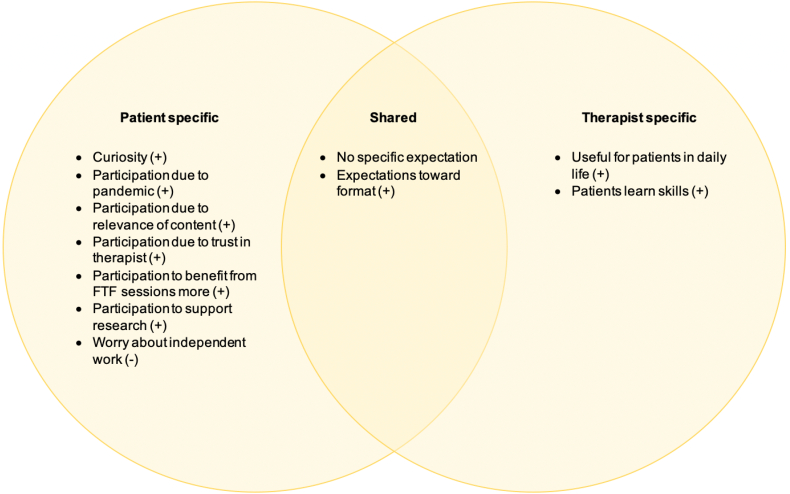
Expectations reported by patients, therapists, and by both. Note. Positive expectations (+), negative expectations (−).

Members of both groups reported having no specific expectations toward BT, and members of both groups also reported positive expectations toward the format. While therapists had no negative expectations toward BT, one patient mentioned worrying about independent work (Fig. 1).

#### 3.3.2 The internet-based program

**Fig. 2:**
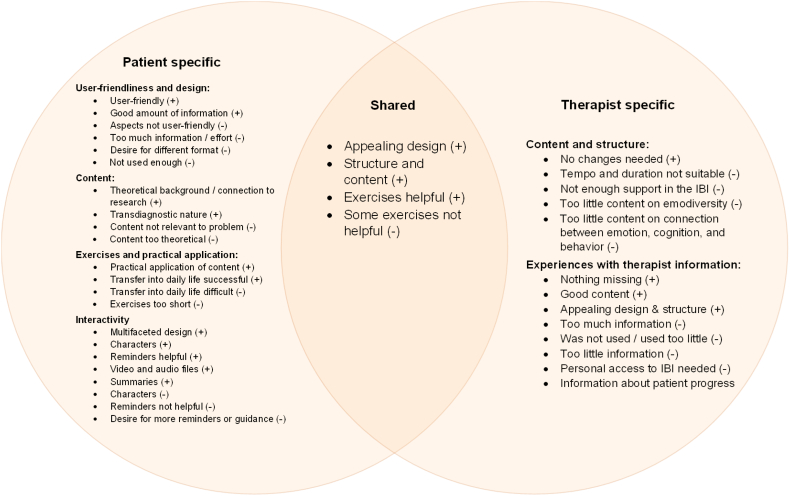
Experiences regarding the internet-based program reported by patients, therapists, and both. Note. Positive experiences (+), negative experiences (−).

Members of both groups mentioned the appealing IBI design, the content and structure as positive, and the exercises as helpful. Members of both groups also mentioned exercises as not having been helpful. While patients commented in more detail on the specificities of the program (content, interactivity, user-friendliness, and design), therapists talked in detail about their experiences with the therapist material (Fig. 2).

#### 3.3.3 Emotion regulation and symptomatology

**Fig. 3:**
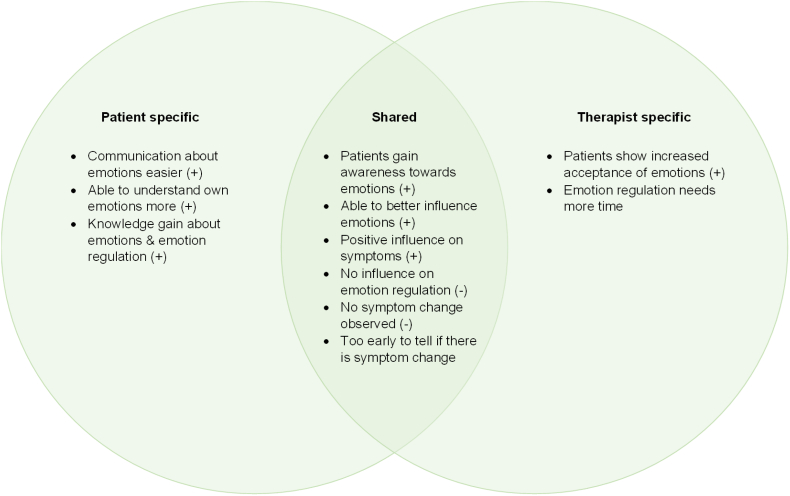
Experiences regarding patient emotion regulation and symptomatology reported by patients, therapists, and both. Note. Positive experiences (+), negative experiences (−).

Regarding positive experiences, members of both groups reported that patients had more awareness of their own emotions and were better able to influence them due to the intervention. Members of both groups also observed a positive influence on symptoms. While therapists noted that patients showed increased acceptance of emotions, patients reported easier communication about emotions, being able to understand own emotions more and having gained knowledge about emotions and emotion regulation. Concerning negative experiences, members of both groups mentioned that no influence on emotion regulation or symptom change was observed (Fig. 3).

#### 3.3.4 The therapeutic relationship

**Fig. 4:**
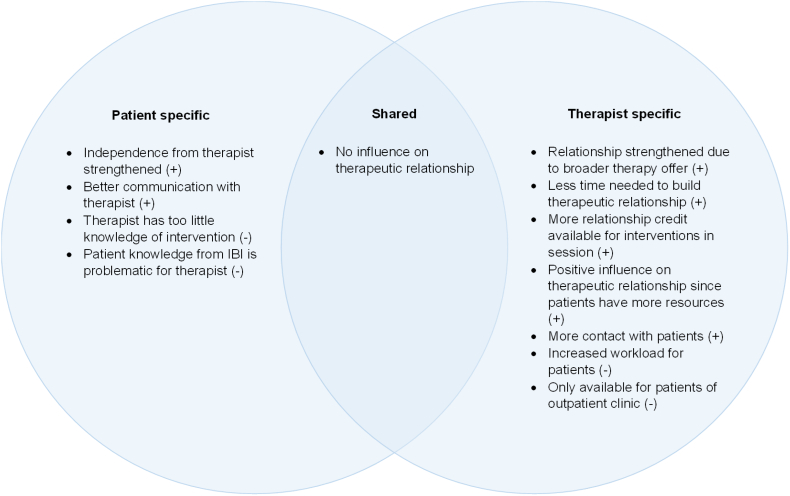
Experiences regarding the therapeutic relationship, reported by patients, therapists, and both. Note. Positive experiences (+), negative experiences (−).

Members of both groups mentioned that the intervention did not influence the therapeutic relationship. Concerning positive and negative experiences, therapists and patients mentioned different aspects, with therapists reporting a broader range of positive experiences than patients (Fig. 4).

#### 3.3.5 The blended format

**Fig. 5:**
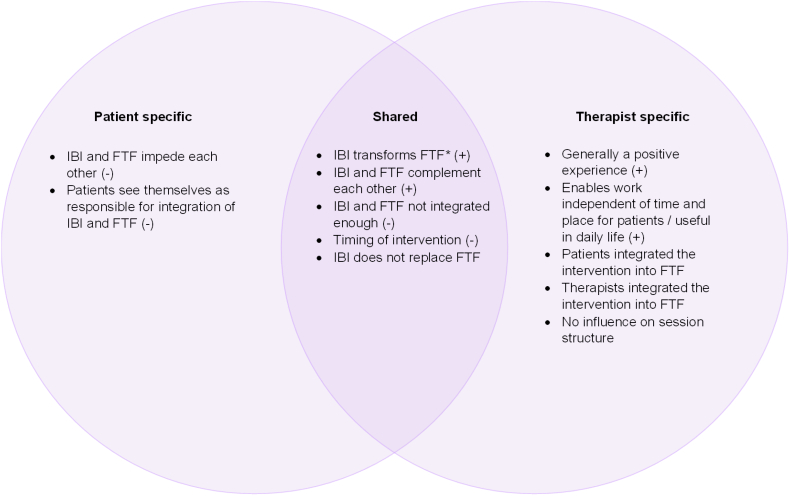
Experiences regarding the blended format reported by patients, therapists, and both. Note. Positive experiences (+), negative experiences (−). *Patients mentioned IBI and FTF transforming each other, whereas therapists mentioned IBI transforming FTF.

Members of both groups felt that the IBI positively transformed FTF sessions and that the IBI managed to complement them. Therapists experienced the blended format positively and noted that it enabled time and place independent work for patients. Regarding negative aspects, members of both groups felt that the IBI and FTF treatment were not integrated enough and that the timing of the intervention within the psychotherapeutic treatment could have been improved. Moreover, patients also felt that the IBI and FTF sessions impeded each other, and some patients felt like they were responsible for integrating the IBI into FTF sessions (Fig. 5).

## 4 Discussion

This study investigated patients' and therapists' experiences with BT focusing on emotion regulation in outpatient psychotherapy. Section 4.1 integrates the principal findings of the study with previous literature and highlights tentative recommendations on how to improve patients' and therapists' experiences concerning treatment expectations, the IBI, symptomatology and emotion regulation, the therapeutic relationship and the blended format.

### 4.1 Principal findings and recommendations

Prior to the intervention, more than 50 % of patients and several therapists (37.5 %) had no specific expectations toward BT, a finding in line with a previous study reporting neutral expectations (Siemer et al., 2020). While therapists further reported only positive expectations, one patient reported fear of independent work. Thus, therapists may want to conduct an introductory FTF session as done in previous BT protocols (e.g., Kooistra et al., 2016), in order to build on expectations and alleviate patient fears.

• Recommendation: Therapists may want to start BT with an FTF session that transforms neutral patient expectations and openly discusses possible apprehensions.

Both groups felt that the design of the IBI was appealing, the structure and content were positive, and that the exercises were helpful. These findings are in line with similar studies (Urech et al., 2019; Etzelmueller et al., 2018). However, patients (50 %) and therapists (25 %) also mentioned that exercises in the IBI were not helpful. Specifically, patients mentioned that certain exercises did not feel personally relevant. Perhaps, in comparison to disorder-specific treatments, transdiagnostic interventions seem less problem-specific and thus less immediately relevant to patients' problems. Therefore, a more individualized, tailored, or modular approach to the transdiagnostic IBI may improve the experience. However, tailoring of the IBI may also be challenging, as modularisation requires indicators of matching between content and individual need (Dalgleish et al., 2020).

• Recommendation: Individualized content may want to be included in transdiagnostic IBI. For example, by providing a variety of exercises that personalize the topic of emotion regulation and that can be chosen depending on patient needs or clinical judgement.

Fifty percent of therapists reported that they would have preferred direct access to the IBI. Fifty percent also reported that they would have wanted access to patient progress. Innovative ways to allow therapists this type of access to internet-based interventions, such as for example, visual analytics dashboards, exist (Grieg et al., 2019).

• Recommendation: Therapists may want to monitor patient progress within the IBI, for example, via technology that enables an innovative and easy to use therapist dashboard.

Patients and therapists reported various positive experiences regarding emotion regulation and symptomatology. For example, patients (50 %) and therapists (37.5 %) reported that patients were more aware of their emotions and better able to influence them. This finding supports the idea that, subjectively, patients and therapists see the benefits of transdiagnostic BT. Interestingly, temporal relationships between intervention, emotion regulation, and symptomatology were also mentioned by participants. After 6 weeks, 25 % of patients and therapists stated that it was too early to tell if there was actual symptom change.

• Recommendation: When interventions target transdiagnostic processes such as emotion regulation, it may be relevant to inform patients that changes regarding symptomatology may not be as immediately visible as changes regarding emotion regulation.

Most patients (75 %) and therapists (62.5 %) experienced no effect of the intervention on the therapeutic relationship. Interestingly, while therapists feared that the increased workload for patients might be problematic for the therapeutic relationship, patients did not mention this. In contrast, patients mentioned the lack of therapist knowledge on IBI content as problematic. Interestingly, in a more recent study on practitioner experiences regarding the working alliance in blended CBT for depression (Doukani et al., 2022), practitioners mentioned low confidence and practice (lack of expertise and experience delivering the intervention) as one barrier to building and maintaining a working alliance.

• Recommendation: Therapists may want to gain in-depth knowledge on IBI content and terminology before blending. Therapists may also not need to worry about increased patient workload being a concern for patients.

Finally, concerning the format, almost all therapists and patients (87.5 % respectively) felt that the IBI and FTF sessions were not integrated enough (see Urech et al., 2019 for similar findings). Perhaps integrated blends are harder to conceptualize when a transdiagnostic intervention is added to disorder-specific therapy as this involves fundamental changes to therapy structure and may add to therapist burden. Interestingly, some patients in the current study felt it was their responsibility to integrate the internet-based program into psychotherapy sessions.

• Recommendation: Transdiagnostic BT may want to attempt an intricate integration of FTF and IBI elements. Integration is not only the responsibility of the patient and this may want to be communicated by the therapist prior to treatment.

Both patients and therapists felt that the IBI positively transformed FTF sessions and managed to complement FTF sessions. For example, both patients and therapists mentioned that therapy could focus on more personal content because the IBI provided psychoeducation. A further aspect mentioned by both, was that the timing of the intervention should have been more flexible within psychotherapy. Similarly, Toonders et al. (2021) reported that autonomy regarding when to stick to the treatment protocol was a problem for healthcare workers providing BT.

• Recommendation: Therapists and patients may want to decide mutually when in the individual therapy process, an IBI is added to FTF psychotherapy.

### 4.2 Strengths and limitations

This study has several limitations. First, the study sample was small and may not be representative for the population of patients and therapists in an outpatient setting. However, the sample size was comparable to other qualitative studies in the field (Patel et al., 2020). Second, not every individual contacted for interview participation agreed to participate. Three out of nine patients who did not agree to participate did not provide post-assessment data. Possibly, participants without post-assessment data had more negative experiences with BT, which could have introduced a selection bias. Third, the therapists' positive attitude toward BT may in part result from the research affiliation of the outpatient clinic. Fourth, interview participants responded to pre-defined questions in the interview guideline. Perhaps, certain aspects that were important to interviewees were not covered in the interview. An attempt to consider this limitation was included in the interview guide with the question “other topics important to participants”. Furthermore, the interview guide explored transdiagnostic elements by asking about the content of the IBI and by asking about effects on the transdiagnostic aspect of emotion regulation. Future studies may want to focus more on the transdiagnostic angle by including more specific questions on the topic. For example, therapists could be asked about their specific expectations toward or attitudes toward transdiagnostic compared to disorder-specific BT. Finally, future studies may also want to consider using a standardized instrument with known topics relevant to BT for their interviews. The results of this and previous studies may help develop such an instrument. This study also has some noteworthy strengths. First, this is the first study that investigated both patient and therapist experiences with the same transdiagnostic BT. Second, the inter-rater agreement, which was assessed with independent raters for both category systems, was sufficiently high. Third, all interviewed patients and therapists had sufficient exposure to the intervention prior to the interview.

## 5 Conclusion

The present study reveals important experiences regarding the delivery of transdiagnostic treatment in a blended format from the patient and therapist perspective. Based on the study results, it seems evident that more research is needed to find an optimal way to integrate face-to-face and internet-based components within one treatment to provide the best possible experience for both patients and therapists.

The following are the supplementary data related to this article.

Supplementary material A Details on patient and therapist samples Supplementary material A

Supplementary material B Interview guides Supplementary material B

Supplementary material C Category systems with anchor examples Supplementary material C

Supplementary material D COREQ Checklist Supplementary material D

## Funding

This research did not receive any specific grant from funding agencies in the public, commercial, or not-for-profit sectors and was entirely funded by the Department of Clinical Psychology and Psychotherapy, 10.13039/100009068University of Bern, Switzerland.

## Declarations

Ethical approval has been obtained by the Cantonal Ethics Committee Bern (ID 2019–01929). Informed consent was obtained from all participants.

## Declaration of competing interest

The authors declare that they have no known competing financial interests or personal relationships that could have appeared to influence the work reported in this paper.
