# Supplementary material for: Two sides of the same coin? Patient and therapist experiences with a transdiagnostic blended intervention focusing on emotion regulation
Source: Internet Interv. 2022 Nov 10;30:100586. doi: 10.1016/j.invent.2022.100586 (PMC9663910; doi:10.1016/j.invent.2022.100586)
Supplement: Supplementary material A — Details on patient and therapist samples [file mmc1.pdf]

Supplementary Material A

Table A.1

Patient Sample Characteristics

Characteristic	Full sample (N = 17)	Interviewed (n = 8)	Not interviewed (n = 9)	Statistic
Age (years): *Mdn*, range	27.00, 18–58	27.00, 23–36	27.00, 18–58	U = 38.50 p = .82
Female gender, *n* (%)	13 (76.5)	7 (87.5)	6 (66.7)	Fisher's Exact Test, p = .58
Primary diagnosis, *n* (%)	AD: 2 (11.8), DE: 3 (17.6), ED: 1 (5.9), GAD: 1 (5.9) RDD: 5 (29.4), PD: 1 (5.9), PSPD: 1 (5.9), SP: 3 (17.6)	AD: 1 (12.5), DE: 1(12.5), ED: 1 (12.5), GAD: 1 (12.5), RDD: 3 (37.5), SP: 1 (12.5)	AD: 1 (11.1), DE: 2 (22.2), RDD: 2 (22.2), PD: 1 (11.1), PSPD: 1 (11.1), SP: 2 (22.2)	
Educational level at university / university of applied sciences level, *n* (%)	11 (64.7)	6 (75.0)	5(55.6)	Fisher's Exact Test, p = .62
Symptom severity at baseline (GSI–BSI), *Mdn*, range	0.85, 0.25–2.89	0.97, 0.62–1.77	0.85, 0.25–2.89	U = 33.00, p = .82
Number of REMOTION modules at T1, *Mdn*, range [a]	5.00, 0–6	6.00, 2–6	3.00, 0–6	U = 9.50, p = .01*
Number of FTF sessions T0 to T1, *Mdn*, range	5.00, 1–13	5.50, 0–6	4.00, 1–13	U = 24.50, p = .28
SUS Score at T1, *Mdn*, range	80.00, 55.00–95.00 [b, d]	83.75, 55.00–95.00	76.25, 65.00–92.50 [c, d]	U = 21.00, p = .76

Characteristic	Full sample ($N = 17$)	Interviewed ($n = 8$)	Not interviewed ($n = 9$)	Statistic
Within-group effect size from baseline to T1 on the BSI–GSI: Cohen's *d* (95% CI)	−0.40 (−1.14–0.35) [b, d]	−0.20 (−1.18–0.78)	−0.58 (−1.74–0.58) [c, d]	
Within-group effect size from baseline to T1 on the DERS: Cohen's *d* (95% CI)	−0.56 (−1.72–0.60) [b, d]	−0.48 (−1.47–0.52)	−0.61 (−1.80–0.58) [c, d]	
Reliable improvement at T1, BSI–GSI (*n*) [e]	2 [b, d]	0	2 [c, d]	Fisher's Exact Test, $p = .17$
Reliable deterioration at T1, BSI–GSI (*n*) [e]	0 [b, d]	0	0 [c, d]	

Note. AD = Adjustment Disorder, DE = Depressive Episode, RDD = Recurrent Depressive Disorder, ED = Eating Disorder, Unspecified, SP = Social Phobia, GAD = General Anxiety Disorder, PD = Panic Disorder, PSPD = Persistent Somatoform Pain Disorder. BSI-GSI= Brief Symptom Inventory – Global Severity Index (Franke, 2000). SUS = System Usability Scale (Brooke, 1996). DERS = Difficulties in Emotion Regulation Scale (Gratz & Roemer, 2004).

[a] a completed module was defined as each main module page having been visited at least once. [b] $N = 14$ as three individuals did not complete post-assessment. [c] $n = 6$ as three individuals did not complete post-assessment. [d] One participant in the not-interviewed group had one missing item on the BSI at T1, one missing item on the DERS at T1 and two missing items on the SUS at T1, these were imputed with the mean score for the specific participant and scale at T1. [e] Reliable change score was calculated based on the formula suggested by Jacobson and Truax (1991) and using re-test reliability (r = 0.90) reported for a sample of psychiatric outpatients described by Derogatis in Franke (2000), *Reliable Change Criterion* = 0.56.

$^{*}p < .05$

Table A.2

Therapist Sample Characteristics

Characteristic	Full sample ($N = 12$)	Interviewed ($n = 8$)	Not interviewed ($n = 4$)	Statistic
Age (years): *Mdn*, range	33.50, 28–44	31.50, 28–35	41.00, 28–44	$U = 25.00$, p =.15
Female gender: *n* (%)	10 (83.3)	6 (75.0)	4 (100.0)	Fisher's Exact Test, $p = .52$
In psychotherapy training: *n* (%)	10 (83.3)	8 (100.0)	2 (50.0)	Fisher's Exact Test, $p = .09$
Years of experience: *Mdn*, range	3.13, 0–14	2.75, 0–5	7.63, 2–14	U =23.00, $p = .28$
Attitude toward online interventions (Scale 1–5, 5 = *very helpful*): *Mdn*, range	4.50, 4–5	4.00, 4-5	5.00, 4-5	$U = 22.00$, $p = .37$
Importance of emotion regulation as a topic in psychotherapy (Scale 1–5, 5 = *very important*): *Mdn*, range	5.00, 4–5	5.00, 4–5	5.00, 5–5	$U = 22.00$, $p = .37$

$^{*}p < .05$. All characteristics reported at baseline-assessment.

Supplementary Material A References

Brooke, J. (1996). SUS—A quick and dirty usability scale. In P. W. Jordan, B. Thomas, I. L. McClelland, & B. Weerdmeester (Eds.), *Usability evaluation in industry* (pp. 4–7). CRC Press. http://doi.org/10.1201/9781498710411-35

Franke, G. H. (2000). *BSI. Brief Symptom Inventory—German Version. Manual.* Beltz.

Gratz, K. L., & Roemer, L. (2004). Multidimensional assessment of emotion regulation and dysregulation: Development, factor structure, and initial validation of the difficulties in emotion regulation scale. *Journal of Psychopathology and Behavioral Assessment*, *26*(1), 41–54. https://doi.org/10.1023/B:JOBA.0000007455.08539.94

Jacobson, N.S., & Truax, P. (1991). Clinical significance: A statistical approach to defining meaningful change in psychotherapy research. *Journal of Consulting and Clinical Psychology, 59*(1), 12–19.
