# Supplementary material for: Two sides of the same coin? Patient and therapist experiences with a transdiagnostic blended intervention focusing on emotion regulation
Source: Internet Interv. 2022 Nov 10;30:100586. doi: 10.1016/j.invent.2022.100586 (PMC9663910; doi:10.1016/j.invent.2022.100586)
Supplement: Supplementary material B — Interview guides [file mmc2.pdf]

## Supplementary Material B

### Table B.1

*The Semi-Structured Interview Guide (Patients). Translated into English from Suter (2020).*

| Topic | Question |
|---|---|
| Expectations / motivation | What motivated you to take part and what were your expectations toward the intervention? [a-c, e-j] |
| General experience | How did you experience the intervention in general? [a, b, h] |
| Internet-based program REMOTION | *User-friendliness:*<br>How did you perceive the user-friendliness of the internet-based program REMOTION? [b, d-f, i]<br><br>Which aspects were helpful, impeding, or missing for you with regards to user-friendliness? [a, j]<br><br>*Content/structure:*<br>How did you perceive the content and the structure of the internet-based program REMOTION? [b, d, e, i]<br><br>Which aspects were helpful, impeding, or missing for you? [a, j] |
| Blended therapy | *Blended therapy:*<br>What was helpful, impeding, or missing for you with regards to the combination of REMOTION and regular psychotherapy? [a, c-h, j]<br><br>*Integration in therapy:*<br>How did you or your therapist integrate the internet-based program into the therapy sessions? [f] |
| Therapeutic relationship | Did this intervention have an impact on the relationship with your therapist and, if so, what kind of impact? [e-g, i] |
| Impact / effectiveness of the intervention | *Changes:*<br>Were you or your surroundings able to notice any changes in you and, if so, what kind of changes? [a, b, d-g]<br><br>*Impact on symptomatology / emotion regulation:*<br>Did the intervention influence your symptoms or emotion regulation and, if so, how? [a, b, d-g] |
| Suggestions for improvement | What kind of changes with regards to the intervention would you suggest? And why? [a, b, e-j]<br><br>Which aspects of the intervention would you adapt, maintain, or omit? [a, j] |
| Open-ended question | Now that we've come to the end of this interview, are there any aspects with regards to this intervention that we haven't addressed yet and that you would like to share with us? [a, j] |

[a] Elliott et al. (2001). [b] Etzelmueller et al. (2018). [c] Fernández-Álvarez et al. (2017). [d] Fleischmann et al. (2018). [e] Lillevoll et al. (2013). [f] Lindgreen et al. (2018). [g] Ly et al. (2015). [h] Schuster et al. (2018). [i] Wallin et al. (2018). [j] Walsh et al. (2018).

**Table B.2**

*The Semi-Structured Interview Guide (Therapists). Translated into English from Walsh (2021).*

| Topic | Question |
|---|---|
| Expectations and experience | What were your expectations toward the intervention? [a] |
| | Did you already have experience with online interventions as an add-on to psychotherapy? [b, c] |
| General experience with the intervention | How did you experience the intervention in general? [a] |
| Therapist material | How did you perceive the therapist material of the intervention? Which aspects of it were helpful, impeding, or missing for you? [c] |
| Blended therapy | How did you or your patient integrate the internet-based program into the therapy sessions? [a, d] |
| | What was helpful, impeding, or missing for you with regard the intervention being blended therapy in comparison with regular psychotherapy? [c, e, f] |
| Therapeutic relationship | Did this intervention have an impact on the therapeutic relationship with your patient? If so, what kind of impact? [a, f, g] |
| Impact of the intervention | Did this intervention change the symptoms or the emotion regulation of the client? If so, how? [f, g] |
| | Did the intervention change your usual structuring of therapy sessions? If so, how? [a, c] |
| Suggestions for improvement | What kind of changes with regards to the intervention would you suggest? And why? [a, c] |
| | Which aspects of the intervention would you adapt, maintain, or omit? [a, c] |
| Open-ended question | Now that we’ve come to the end of this interview, are there any aspects with regards to this intervention that we haven’t addressed yet and that you would like to share with us? [a, c] |

[a] Wilhelmsen et al. (2014). [b] Titzler et al. (2018). [c] Mol et al. (2020). [d] Rodda et al. (2019). [e] Schuster et al. (2019). [f] van der Vaart et al. (2014). [g] Urech et al. (2019).

**Supplementary Material B References**

Elliott, R., Slatick, E., & Urman, M. (2001). Qualitative change process research on psychotherapy: Alternative strategies. *Psychologische Beiträge, 43*(3), 69. https://search.proquest.com/scholarly-journals/qualitative-change-process-research-on/docview/212158667/se-2?accountid=17231

Etzelmueller A., Radkovsky A., Hannig W., Berking M., & Ebert D. D. (2018) Patient's experience with blended video- and internet based cognitive behavioural therapy service in routine care. *Internet Interventions, 12*, 165–175. https://doi.org/10.1016/j.invent.2018.01.003

Fernández-Álvarez, J., Díaz-García, A., González-Robles, A., Baños, R., García-Palacios, A., & Botella, C. (2017). Dropping out of a transdiagnostic online intervention: A qualitative analysis of client's experiences. *Internet Interventions, 10*, 29–38. https://doi.org/10.1016/j.invent.2017.09.001

Fleischmann, R. J., Harrer, M., Zarski, A. C., Baumeister, H., Lehr, D., & Ebert, D. D. (2018). Patients' experiences in a guided internet- and app-based stress intervention for college students: A qualitative study. *Internet Interventions, 12*, 130–140. https://doi.org/10.1016/j.invent.2017.12.001

Lillevoll, K. R., Wilhelmsen, M., Kolstrup, N., Høifødt, R. S., Eisemann, M., & Risør, M. B. (2013). Patients' experiences of helpfulness in guided internet-based treatment for depression: Qualitative study of integrated therapeutic dimensions. *Journal of Medical Internet Research, 15*(6), e126. https://doi.org/10.2196/jmir.2531

Lindgreen, P., Lomborg, K., & Clausen, L. (2018). Patient experiences using a self-monitoring app in eating disorder treatment: Qualitative study. *JMIR mHealth and uHealth, 6*(6), e10253. https://doi.org/10.2196/10253

Ly, K. H., Janni, E., Wrede, R., Sedem, M., Donker, T., Carlbring, P., & Andersson, G. (2015). Experiences of a guided smartphone-based behavioral activation therapy for depression: A qualitative study. *Internet Interventions, 2*(1), 60–68. https://doi.org/10.1016/j.invent.2014.12.002

Mol, M., van Genugten, C., Dozeman, E., van Schaik, D. J., Draisma, S., Riper, H., & Smit, J. H. (2020). Why uptake of blended internet-based interventions for depression is challenging: A qualitative study on therapists' perspectives. *Journal of Clinical Medicine, 9*(1), 91. https://doi.org/10.3390/jcm9010091

Rodda, S. N., Merkouris, S., Lavis, T., Smith, D., Lubman, D. I., Austin, D., Harvey, P., Battersby, M., & Dowling, N. A. (2019). The therapist experience of internet delivered CBT for problem gambling: Service integration considerations. *Internet Interventions, 18*, 100264. https://doi.org/10.1016/j.invent.2019.100264

Schuster, R., Sigl, S., Berger, T., & Laireiter, A. R. (2018). Patients' experiences of web-and mobile-assisted group therapy for depression and implications of the group setting: Qualitative follow-up study. *JMIR Mental Health, 5*(3), e49. https://doi.org/10.2196/mental.9613

Schuster, R., Kalthoff, I., Walther, A., Köhldorfer, L., Partinger, E., Berger, T., & Laireiter, A. R. (2019). Effects, adherence, and therapists' perceptions of web-and mobile-supported group therapy for depression: Mixed-methods study. *Journal of Medical Internet Research, 21*(5), e11860. https://doi.org/10.2196/11860

Suter, J. (2020). Patients' perspectives on positive, negative and improvement aspects of REMOTION, a blended, transdiagnostic intervention: A qualitative content analysis [Master Thesis]. Institute of Psychology, University of Bern, Switzerland.

Titzler, I., Saruhanjan, K., Berking, M., Riper, H., & Ebert, D. D. (2018). Barriers and facilitators for the implementation of blended psychotherapy for depression: A qualitative pilot study of therapists' perspective. *Internet Interventions, 12*, 150–164. https://doi.org/10.1016/j.invent.2018.01.002

Urech, A., Krieger, T., Möseneder, L., Biaggi, A., Vincent, A., Poppe, C., Meyer, B. Riper, H., & Berger, T. (2019). A patient post hoc perspective on advantages and disadvantages of blended cognitive behaviour therapy for depression: A qualitative content analysis. *Psychotherapy Research, 29*(8), 986–998. https://doi.org/10.1080/10503307.2018.1430910

van der Vaart, R., Witting, M., Riper, H., Kooistra, L., Bohlmeijer, E. T., & van Gemert-Pijnen, L. J. (2014). Blending online therapy into regular face-to-face therapy for depression: Content, ratio and preconditions according to patients and therapists using a Delphi study. *BMC Psychiatry, 14*(1), 355. https://doi.org/10.1186/s12888-014-0355-z

Wallin, E., Norlund, F., Olsson, E. M. G., Burell, G., Held, C., & Carlsson, T. (2018). Treatment activity, user satisfaction, and experienced usability of internet-based cognitive behavioral therapy for adults with depression and anxiety after a myocardial infarction: Mixed-methods study. *Journal of Medical Internet Research, 20*(3), e9690. https://doi.org/10.2196/jmir.9690

Walsh, N. (2021). Eine qualitative Inhaltsanalyse der transdiagnostischen Blended Therapie REMOTION: Positive und negative Aspekte, sowie Veränderungsvorschläge aus TherapeutInnensicht [Master Thesis]. Institute of Psychology, University of Bern.

Walsh, S., Szymczynska, P., Taylor, S. J., & Priebe, S. (2018). The acceptability of an online intervention using positive psychology for depression: A qualitative study. *Internet Interventions, 13*, 60–66. https://doi.org/10.1016/j.invent.2018.07.003

Wilhelmsen, M., Høifødt, R. S., Kolstrup, N., Eisemann, M., Chenhall, R., & Risør, M. B. (2014). Norwegian general practitioners' perspectives on implementation of a guided web-based cognitive behavioral therapy for depression: A qualitative study. *Journal of Medical Internet Research, 16*(9), e3556. https://doi.org/10.2196/jmir.3556
