# Supplementary material for: Two sides of the same coin? Patient and therapist experiences with a transdiagnostic blended intervention focusing on emotion regulation
Source: Internet Interv. 2022 Nov 10;30:100586. doi: 10.1016/j.invent.2022.100586 (PMC9663910; doi:10.1016/j.invent.2022.100586)
Supplement: Supplementary material C — Category systems with anchor examples [file mmc3.pdf]

Supplementary Material C

Table C.1

Patient Experiences with the Blended Intervention (Category System)

Main theme<br>*Subtheme*	*N* (%) coded, no. of segments	Definition	Supporting quotation (patient number)
Expectations/reasons for participation			
No specific motivation or expectation	5 (62.5) 6	No expectations or specific motivation for participation.	*Uh, there was nothing that motivated me REALLY, but uh, I was asked openly if I wanted to take part. (P_2)*
Participation to support research	4 (50) 5	Participation due to wish to support research.	*Because // research interests me. (P_3)*
Participation due to relevance of content / expectations toward content	3 (37.5) 6	Participation due to positive attitude toward content, relevant of content or positive expectations toward content.	*Because I was always overwhelmed by my extreme emotions. And I explained this during a talk with my therapist, and they explained and recommended the, uh, study to me (.) And that sounded very useful. (P_5)*
Participation due to expectations toward format	2 (25) 5	Positive expectations toward / participation because of format.	*Uh, // on the one hand uh, that I can work from home. Also, that I don't, uh, need to wait for the weekly appointments or whichever frequency I have to wait for psychotherapy for, but can always work on content when I feel like it. (P_3)*
Curiosity	2 (25) 4	Curiosity mentioned as reason for participation.	*Exactly. And I/ I didn't really know/if this program, uh, yes, would even cover a need I had (.) I was actually mainly curious and thought I want to take every opportunity I can get. (P_7)*
Participation to benefit from FTF sessions more	2 (25) 3	Participation due to hope to benefit more from FTF sessions through intervention.	*Yes, I was motivated by (.) uh, I thought that if I do psychotherapy (.) I want to benefit from it as much as possible. (P_7)*
Worry about independent work	1 (12.5) 1	Worry or apprehension regarding independent work with intervention.	*Well, I, uh, well I thought (...) well because I had the feeling, that if one has to work so independently, that that wouldn't be, uh, so successful. (P_2)*
Participation due to pandemic	1 (12.5) 1	Participation due to reasons connected to pandemic.	*Especially in times of a pandemic (inaudible) it seemed very sensible. (P_5)*
Participation due to trust in therapist	1 (12.5) 1	Participation due to trust placed in therapist.	*And I decided to take part without really thinking about it. Because I simply trusted the word, uh, of my therapist. (P_2)*
IBI: User-friendliness and design			
Aspects not user-friendly	7 (87.5) 20	Patients report difficulties or missing elements regarding user-friendliness (saving of material, login, viewing user inputs, switching between pages, module names etc.).	*Well, with regard to adaption, it would be what I've already mentioned a few times now, the names of the modules, applying or implementation or uh, something, well the terminology of the modules really. (P_2)*
User-friendly	7 (87.5) 22	Patients report positive aspects with regard to usability (easy to use, user-friendly, good module overviews etc.).	*It made sense, that already in the introduction, uh, well where everything was introduced, how everything works, that it was already quite interestingly structured, I then really worked through everything and was then actually familiar with all the uh, applications. (P_3)*
Desire for a different format	5 (62.5) 23	Patients report desire for paper version of the material, offline access and/or APP instead of internet-based program.	*And I would've, uh found it helpful, if, if there would've been like a (.) separate sheet of paper, where ONLY the answers are visible. (P_1)*

Main theme<br>*Subtheme*	*N* (%) coded, no. of segments	Definition	Supporting quotation (patient number)
Too much information / effort	4 (50) 7	Patients report the program contains too much information (text) or is too effortful.	*Okay. Yes, well particularly the first part with the information, that was very good and helpful. But then at the end, particularly the session on applying and practicing was too broad for me (.) and also too, uh (.) well the effort was somehow, which one has to individually put in, was just too much for me and I've never really (.) found the time to engage with it. (P_6)*
Appealing design	3 (37.5) 6	Patients report on appealing design of the program (colors, pictures, progress bars, quotes etc.).	*Well, I was uh, at first rather surprised, because uh, I uh (laughter) because it is so colorfully and creatively designed, well purely from a visual standpoint. (P_3)*
Not used enough	3 (37.5) 6	Patient report not having used the program enough or wish they had used it more.	*I actually think that it was good the way it was. It is a little bit like, really a good application, there is very little that bothered me. The only aspect would be that I should have done more with it. But this is not the fault of the study but rather my own. Exactly. (P_6)*
Good amount of information	2 (25) 5	Patients report that the amount of information provided in the program is positive.	*For me, well it was interesting, but it was not too much, that I didn't feel like it anymore. (P_2)*
IBI: Content			
Content & structure of content interesting	7 (87.5) 31	Patients report the content and structure of the content is positive.	*Uh it was uh, nice to first learn something about the general topic. And then the identification, selection and implementation - I actually think that it was logically structured. (P_3)*
Content too theoretical	2 (25) 4	Patients report the content is too theoretical in nature.	*Yes, I (.) It was good that, even though it was rather intellectually demanding, that the topic of emotions was broken down a little. I was able to follow everything as I was reading it.* [...] *but I could/have imagined, yes, that it could be that for other people* [...] *it might be difficult to understand everything, the technical terms and the footnotes and the studies. And so on. It was quite/ despite everything, I felt it was quite a scientific jargon. I noticed that. (P_7)*
Theoretical background / connection to research positive	2 (25) 3	Patients mention the research background/theoretical foundation of the program as a positive aspect.	*Uh, I, found the structure to be very good. That, well what I also appreciated was, that uh, first a little bit of, uh, theoretical uh, scientific background knowledge was provided. (P_1)*
Transdiagnostic nature positive	1 (12.5) 1	Patients mention positive experiences with transdiagnostic content.	*Uh, I have to think about this for a moment, (4) uh, (4) yes, it was certainly helpful, uh, that, I think, that REMOTION, how I (.) perceived it, was actually very practically oriented. And did not focus so much on the mental health disorder, well the type of the mental health disorder, but. (P_3)*
Content not relevant to specific problem	1 (12.5) 1	Patients report that emotion regulation is not their main problem and content thus not relevant.	*Furthermore, I doubt that it is my main problem, dealing with emotions and I think that if someone really has difficulties with that, it would have fit this person more than it fit for me…And I uh, didn't in that sense search for this particular type of intervention. (P_7)*
IBI: Interactivity			
Multifaceted design	4 (50) 11	Patients report that the IBI is multifaceted (various channels, elements, exercises, audio files, videos, etc.).	*Uh, it was nice, that it uh, different uh, channels were used. Well, uh, t/the quotes and uh, text and videos and exercises. (P_3)*
Videos and audio files positive	4 (50) 5	Videos and audio files are rated positively.	*Uh, I think it was very good with the videos. Uh, I don't really l/like reading long texts when I'm, uh, participating in a study like this. And it was very interactive, well the videos were, uh, w/were a hi/highlight for me. (P_4)*

Main theme<br>*Subtheme*	*N* (%) coded, no. of segments	Definition	Supporting quotation (patient number)
Reminders helpful	3 (37.5) 6	E-mail reminders/contact with the study team/mails are perceived positively.	*And in that instance the mail was, uh, very helpful for me, also the phrasing in the mail. (P2)*
Characters positive	3 (37.5) 4	Characters are described positively.	*And I (.) of course appreciated, that also the/ well I think it was important that there were these examples with the two characters. This was certainly helpful, uh, for understanding it in everyday life (.) to transfer it into everyday life. (P7)*
Desire for more reminders and or guidance	3 (37.5) 6	Desire for more guidance within the program (e-mails to interact with the study team).	*As a reminder to do more with the program. I probably would need/ me personally I would probably need more reminders or something like that, so that I really work on it for a little bit every day. And that I don't work through it once, do it and then forget about it for a week until next time where I think oh (..) today I need to finish the second part. But that's exactly how it is in therapy too. But it is difficult, I couldn't tell you how to do it better. (P5)*
Reminders not helpful	2 (25) 3	Reminders / contact with the study team not helpful / generate pressure or negative feelings.	*Uh, this would be my wish, and this other thing. Uh, yes, the other negative thing I've written down were uh, the reminders. (P3)*
Characters negative	1 (12.5) 2	Characters in the program are described as negative.	*Uh, (..) I don't know if I always liked the examples with/ the ones with Emma and Alex. (P2)*
Summaries positive	1 (12.5) 2	Summaries are rated positively.	*Uh, I think it was good that it was possible to write summaries. (P2)*
IBI: Exercises and practical application			
Exercise content not helpful/too difficult	4 (50) 6	Exercises are described as not helpful / too effortful or too difficult.	*Uh, yes and just, no that's maybe, not really, well certain exercises, but more so because they were more difficult, because they weren't as relevant for me, but this is (.) different for everyone, I guess. (P2)*
Exercises too short or too little	4 (50) 8	Exercises too short/not enough exercises available / desire for more exercises.	*And uh, (smiles) yes, (..) I don't know if maybe there could be, (.) uh, even more exercises added. (P3)*
Transfer into daily life difficult	3 (37.5) 3	Transfer into daily life missed or not successful.	*That I also (.) I never came across situations where I was able to apply the different exercises on emotion regulation (.) Or where I could determine where I am on the emotion barometer and which strategy I can apply, like it was theoretically proposed. Well, I, somehow, well wasn't really able to implement that. (P6)*
Transfer into daily life successful	2 (25) 3	Contents of the intervention were applied / used in daily life.	*Uh, yes, I, uh, applied the intervention or the tips from the study, a few times. (P3)*
Exercises helpful	1 (12.5) 1	Exercises are described positively.	*Missing, uh, I was given a lot of suggestions on how I could complete the exercises. And two or three times (.) a meditation video. And I've applied these and it was helpful, this was something that was concrete. That I could directly apply. (P7)*
Practical application of content positive	1 (12.5) 1	Practical application is described positively.	*Uh, I have to think about this for a moment, (4) uh, (4) yes, it was certainly helpful, uh, that, I think, that REMOTION, how I (.) perceived it, was actually very practically oriented. And did not focus so much on the mental health disorder, well the type of the mental health disorder, but. (P3)*
Emotion & emotion regulation			
More awareness toward own emotions	4 (50) 5	Patients report having more awareness toward own emotions or recognizing them better.	*Yes, I, I, I, (.) maybe I'm/ because of the intervention I'm more sensitized to it, or I'm more aware in which situations I'm experiencing which emotions. Or maybe also, in situations (.) uh, where I think that I somehow want to change the way I experience my emotions, that I'm like a little bit more sensitized to it, I really think that. (P1)*

Main theme<br>*Subtheme*	*N* (%) coded, no. of segments	Definition	Supporting quotation (patient number)
Able to better influence own emotions	4 (50) 11	Patients report that they can better regulate / influence emotions.	*Uh//, (..) by influencing which type of music I'm listening to, or something like that. Uh, I was able to regulate my emotions better. (P_2)*
Knowledge gain about emotions & emotion regulation	4 (50) 5	Patients report having gained knowledge about emotions in general (not specifically about their own emotions).	*And uh, it was also, well I had the feeling that it reassured me, and I've learned a lot. (P_3)*
No influence on emotion regulation	2 (25) 3	Patients report that there has been no influence on emotion regulation.	*I was not able to see that, no. (P_7)*
Able to understand own emotions more	2 (25) 6	Patients report that they can understand own emotions more.	*Well that I understand, which emotions I have and because of the clear/ well, because of the clear information, how feelings or emotions manifest themselves, psy/ uh well also psychologically and, and in relation to the mood, well. (P_2)*
Communication about emotions easier	1 (12.5) 1	Patients report that they can communicate better about their own emotions.	*Or// that I was also able to better respond, to sort my emotions and speak about them. (P_2)*
Social environment			
No change noticed by social environment	5 (62.5) 5	Social environment did not notice or report a change in the patient.	*And uh, yes, and uh, yes from my social environment I didn't get any direct feedback. (P_3)*
Positive change noticed by social environment	3 (37.5) 3	Positive influence of the intervention was noticed by the social environment.	*Uh, well ME certainly. I don't know if, yes, I think I did receive a comment from my social environment too. But ME in particular uh, I noticed it a lot, yes through these exercises which seemed simple at first glance, but a/ I somehow kept it in the back of my head and uh, in daily life I recalled them from time to time and uh, this has helped me somehow to manage certain situations, or uh, or somehow the awareness that something is happening in my head, or yes uh, I, uh, have noticed this effect. (P_4)*
Positive influence on social interactions	1 (12.5) 2	Paid more attention to the social environment / to other people / positive influence on others.	*Yes. And I have the feeling, that it has also influenced my relationships, because I (..) can react (..) in a more positive manner because of the intervention. (P_2)*
Symptomatology			
Positive influence on symptoms	4 (50) 14	Positive influence on symptoms noticed.	*Uh, (breathing in) uh, I/ there are/ techniques, how one can, uh, regulate emotions and uh, because I have, uh, discovered them, I tried to apply them. And uh, I have the feeling, that this has reduced my symptoms. I think. (P_4)*
No symptom change observed	3 (37.5) 6	No change in symptoms due to the intervention including changes in behavior.	*No, I don't think so. (P_6)*
Too early to tell if there is symptom change	2 (25) 3	Patients report that it is too early to notice an influence on symptoms.	*I think, yes, it probably takes a long time for them to disappear or really (.) improve a lot. (P_3)*
Therapeutic relationship			
No influence on therapeutic relationship	6 (75) 6	No influence on therapeutic relationship noticed.	*No, actually not at all. (P_1)*
Therapist has too little knowledge of intervention	3 (37.5) 4	Patients report that therapists have too little knowledge about the intervention.	*Uh (...) it was interesting, that I / Well I think that the therapists don't really know EXACTLY what's in REMOTION and when I started, in part, talking about it, that helped me figure out, what I understand and what I don't understand yet. (P_2)*
Better communication with therapist	1 (12.5) 4	Better communication with the therapist possible due to the intervention.	*Well, what was helpful as well, was, that because of REMOTION and because I received this background information about emotions, I had the feeling that I was better able to express myself, in therapy, how I felt d/ during the week. (P_2)*

Main theme<br>*Subtheme*	*N* (%) coded, no. of segments	Definition	Supporting quotation (patient number)
Independence from therapist strengthened	1 (12.5) 2	More independence from therapist.	*Depending on/ I think that it, perhaps, has made me somewhat more independent. (P_3)*
Patient knowledge from IBI is problematic for therapist	1 (12.5) 2	Patients assume that knowledge gain from IBI has a negative influence on the therapist.	*Uh, well I/ I know, but like I said, I had the impression that uh, it was perhaps a little bit of a disturbance for the therapist, that I already gained knowledge, but perhaps I'm misjudging this. (P_4)*
Blended format			
IBI and FTF not integrated enough	7 (87.5) 23	Patients report that the two elements are not integrated enough and/or that they wish for more integration.	*But actually, we didn't really talk about it (the intervention) in therapy. (P_1)*
IBI and FTF transform each other	6 (75) 13	IBI and/or FTF therapy transform each other through the combination (example: content etc. is modified/specific content is being talked about/some things are being addressed more or less because of the combination).	*More// uh, actually, in daily life. And in this way uh, I was able to split it up a little bit with psychotherapy, that we were able to go into detail more about personal uh, things there, which weren't covered in REMOTION. (P_3)*
IBI and FTF complement each other	4 (50) 10	Due to the combination of the two formats, there is something added to therapy. But there is no change in IBI or FTF therapy itself.	*For me, uh, REMOTION was somehow like an addition, that certainly is helpful and uh, uh, REMOTION has given me some inputs. (P_1)*
IBI and FTF impede each other	3 (37.5) 4	The IBI and the FTF psychotherapy influence each other negatively or the integration of the two formats is perceived negatively.	*Next to the weekly sessions, taking part in the study was just too much. (P_8)*
IBI does not replace FTF	2 (25) 6	IBI does not replace FTF therapy.	*But (..) uh, without (...) I, well I, for ME, I think WITHOUT the therapy uh, (..) REMOTION wouldn't have shown such an effect. (P_1)*
Wrong timing of intervention	2 (25) 4	Timing of intervention was wrong (should have been used earlier or later).	*Yes, I think that this/ the REMOTION program would make more sense if I wouldn't be at the be/beginning of my therapy. Because in the beginning phase the topic was mainly about which problems I'm facing in daily life, uh, where I want, uh, to improve, what my goals are. Well, there was just a lot to unpack at first. Uh/ and/ the actual therapeutic process hadn't really started yet, and it was more about unpacking everything for the therapist. And (.) because of that, there wasn't any actual support happening in therapy that would have complemented REMOTION at that point. (P_7)*
Patients see themselves as responsible for the integration of IBI and FTF	2 (25) 5	Patients see themselves as responsible for the integration of FTF therapy and IBI or apologize for the lack of integration between the two.	*Uh, missing. (...) At one point the program requested that I should, I think, bring a drawing with me to the therapist. There wasn't really a place for that in therapy. And on the other hand, they never really addressed it either, the program. Perhaps I should've brought it up. Yes, this might have been missing, that, uh, I didn't tell them, or didn't request it enough, or that they weren't informed – I don't know. Well, the cooperation I mean. (P_7)*

Note. To further ensure anonymity, he/she was substituted with the patient/they/them in the quotations and the sentence structure was adapted accordingly. Equivalent patient and therapist numberings in the category system do not indicate a psychotherapy dyad (e.g. T_1 was not the therapist of P_1 etc.). The category system was translated into English from German.

Table C.2

Therapist Experiences with the Blended Intervention (Category System)

Main theme<br>*Subtheme*	N (%) coded, no. of segments	Definition	Supporting quotation (therapist number)
Expectations/reasons for participation			
Useful for patients in daily life	4 (50) 4	Therapists describe the expected usefulness and transfer into daily life/outside of FTF sessions.	*Uh. I thought that the patients are really like, accompanied between the therapy sessions and that they get inputs about emotion regulation in their daily life so that they can also practice more, that it's not just this one hour therapy session a week that is accompanying the patient, but that it is also possible to practice more in daily life. That was my uh expectation. (T_3)*
No specific expectations	3 (37.5) 3	Therapists have no expectations for participation.	*Uh, none (laughter). I didn't have any expectations toward the intervention. (T_8)*
Positive expectations toward format Complements FTF	3 (37.5) 3	Therapists expect the intervention to complement FTF sessions.	*And uh yes, I thought it might be a good/ good complement. That allows specific addressing of the topic of emotions. (T_5)*
Positive expectations toward format Being able to outsource elements of FTF	2 (25) 2	Therapists have positive expectations toward format because elements can be outsourced from FTF or don't have to be discussed in FTF sessions.	*Uh (.) uh my expectations toward the intervention. (.) Uh I (.) think my expectations were that some psychoeducative basics are delivered in the IBI and that I don't need to mention them in FTF (.) a little bit. Well, that uh (.) that's something that takes a lot of time during therapy and uh, I saw, okay there there are some elements of psychoeducation included in the IBI. (.) And well I, I (.) thought that I can reduce the amount of psychoeducation a little bit in FTF. (.) And it is something that also guides the patients between the therapy sessions, (.) uh that they work on the topic emotion regulation, so that there is more room for other (.) other topics within the therapy sessions. (..) Yes, I think (..) that's about it. (T_2)*
Patients learn skills	2 (25) 2	Therapists expect patients to learn new skills.	*Uh. (.) Well I uh thought that the patients or the/ well the study participants uh yes learn emotion regulation skills uh and practice them. Exactly. And embed them into their daily routines. (T_4)*
Previous experiences with BT			
No previous experience	6 (75) 6	Therapists describe having no previous experience with BT.	*None. No experience at all. (T_7)*
Disorder specific experiences	2 (25) 2	Therapists describe having previous experience with disorder-specific BT.	*I've already recommended it to patients two or three times. For example, for sleep disorders. (..) And for eating disorders. (T_4)*
IBI: Exercises			
Exercises not helpful	2 (25) 3	Therapists report that one or more exercises were not helpful for patients.	*Uh, uh (thoughtful). (4) I probably wouldn't, well regarding the content (.) I wouldn't leave out (.) or change anything. (...) Uh maybe uh well the audio exercises, (..) that there should be multiple speakers, who are for example guiding breathing exercises or meditations. (.)* [...] *People have different preferences for different voices* [...] *(.) Uh, so maybe more options with regard to the voices, but that's a detail. (..) Other than that there is nothing else coming to my mind. (T_2)*
Exercises positive/helpful	1 (12.5) 1	Therapists report that the exercises in the program were positively received.	*...and also the exercises. Really important. I absolutely would keep them (6). (T_6)*

Main theme *Subtheme*	N (%) coded, no. of segments	Definition	Supporting quotation (therapist number)
IBI: Content and structure			
Structure positive	5 (62.5) 5	The structure of the program is reported as a positive aspect.	*Yes. (.) I definitely would continue with it. With the patients. I also think that it is good. I think that it is structured in a modular way (clears throat). (.) Uh I think that's a good thing, that (incomp.) it can be done in several steps. (T_6)*
Thematic focus positive	4 (50) 5	The thematic focus of the program is reported as a positive aspect.	*The information content about emotions, where where patients can acquire knowledge, is very good. (T_6)*
No changes needed	4 (50) 7	With regard to the content of the program, no changes are needed.	*Uh. (9) Uh I thi/ Well generally I think/ (4) Yes it it actually, I don't know what could be done differently. I think that it is good, it is good also with regard to the content, everything is included. (.) Uh. Yes. (.) I don't have any suggestions. (T_4)*
Tempo and duration not suitable	2 (25) 3	Therapists report that the tempo or duration of the program seems unfitting or in need of change.	*Good question uh (..) the ONLY thing I've thought about is, if the/ the first module or the introduction I'm not sure uh if it needs to last for one week or if it would be possible to shorten it a bit, well like/ (.) I think that/ yes, the introduction exactly, if it really needs to last for a week or if it could be shortened. That it could be more like background information that one can read and then immediately start. Well, I/ Perhaps this is quite an individual answer, because I think I would want to start immediately after getting the (.) the introductory information. (.) And I think this is perhaps something that could be changed. (T_3)*
Not enough support in the IBI	2 (25) 3	Therapists report too little support for patients within the program during program use.	*Yes, I think the the patients they are doing these exercises for the study. (.) And this may go well. But I think in comparison to the FTF therapy, when they're not getting on with the exercises or notice that they are overwhelmed, then in that moment they don't have anyone they can talk to. And they're sort of on left on their own* [...]. *(T_6)*
Too little content on emodiversity	1 (12.5) 2	Therapists would like more content on emodiversity.	*Uh well I think that for example it would be interesting uh the aspect of (.) uh what is it called? Well e/ emodiversity. It's like emotional diversity. I think that this could be added for example. Well, I'm not sure, but according to this/ according to this/ this information sheet, which I received uh I'm not sure if that was included. Well for example/ There are questionnaires and various uh other options on how that could be included. Well, how the diversity of emotions could be learned. I would have, for example added that. (T_4)*
Too little content on connection between emotion, cognition, and behavior	1 (12.5) 1	Therapists would prefer more content specific to the relationship between emotions, thoughts and behavior.	*Uh, good question. What is missing? Uh (thoughtful). (4) I cannot tell you. Because when it comes to the topic of emotions there's a lot that you can cover but you have to break it down somehow. (.) I don't know. What I/ What I personally use a lot in psychotherapy is the connection between behavior, thoughts, emotions. Well, this triangle. (T_8)*
IBI: Appealing design	3 (37.5) 3	The design of the program is described as positive by therapists.	*Uh the graphic design of the app or this online uh interface was actually very uh very nice. Aesthetically pleasing. Well, I can imagine if I was the patient myself, I would have a lot of joy taking part or w/ working with it. (T_4)*

Main theme<br>*Subtheme*	N (%) coded, no. of segments	Definition	Supporting quotation (therapist number)
Emotion & emotion regulation			
Patients gain awareness toward emotions & emotion regulation	3 (37.5) 6	Therapists report that patients gained awareness toward emotions and emotion regulation due to the intervention.	*Uh well I certainly have the feeling that there is a lot more awareness toward emotions and why (.) they're important. And the person is also more mindful about their emotions. (T_1)*
Patients learn new strategies for emotion regulation/influence own emotions better	3 (37.5) 5	Therapists report that patients have learned new strategies for emotion regulation and/or can influence own emotions better.	*Uh (.) I think, that uh the patient has changed their emotion strategy/ emotion regulation strategy. (.) Uh like uh developed new strategies. Uh for example to change the situation, uh where the patient/ that the patient wasn't really aware of this before, that this could be an option for them. And uh the patient then became aware of this and was able to practice it DIRECTLY in their daily life. Uh (.) it's not that they completely internalized it. But it's definitely a first step toward a/ a change of emotion regulation strategies on behalf of the patient, which was promoted by the intervention. (T_7)*
Emotion regulation needs more time	3 (37.5) 3	Therapists report that patients need more time for emotion regulation (including understanding of own emotions) or that this is still an ongoing topic in therapy.	*Uh the understanding of own emotions (.) would probably need more (.) uh time and work. But I have the feeling that because the patient has dealt with this topic again and again also in between sessions/ (.) this has brought it into focus and this is uh really valuable, to work on it further, because the patient can now tell (.) more often w/ when there's nothing there (.) or uh also found like an access to the body and also an understanding somehow, of how emotions (.) can present in different ways. Well in this regard uh (.) I would say that it has uh (.) initiated, (.) a lot that can be worked on more thoroughly in the future, yes. (T_1)*
No influence on emotion regulation	2 (25) 2	Therapists report that no influence on emotion regulation was perceived or that no judgement can be made.	*I really couldn't say anything reliable about that now because we didn't really speak about emotion regulation to such a degree. (.) Because of this I would probably say no, if I had to choose one direction, but only because the intervention couldn't be implemented enough. (T_3)*
Patients show increased acceptance of emotions	1 (12.5) 1	Therapists report that patients show increased acceptance of own emotions.	*I think that it has certainly uh helped. The patient had a hard time accessing their own emotions or admitting that they are allowed to have emotions. Uh in this regard it has helped a lot. Teaching that it's okay to have emotions* [...] *In that regard it really brought on a change of the patient's problem. (T_8)*
Therapist information			
Therapist material Good content	6 (75) 8	Therapists report that the content of the therapist material is good/informative.	*Well, it was/ I think that it was narrowed down to the basics, and I liked that some practical examples were included. Well, techniques, some of which I already knew, but which were really helpful. (T_4)*
Therapist material Appealing design and structure	5 (62.5) 7	Therapists report that the therapist material has an appealing design or layout/structure.	*Uh in general it was very appealing. Well, the therapist material/ well, there was also a printed version of it. I've looked at it at the beginning and I thought that the structure (.) made a lot of sense and the layout was also appealing and clear. (T_1)*
Therapist material Too little information	5 (62.5) 8	Therapists report that the therapist material could be longer or have more content.	*Missing. (.) Uh. (.) Yes well maybe I uh well the manual or whatever it is called, the exercise sheet that I have received, this uh this I imagine that it could be a bit more detailed or that there could be a little bit more uh uh (..) almost like techniques in a manual included, that I can actually use more in my sessions. (T_4)*

Main theme<br>*Subtheme*	N (%) coded, no. of segments	Definition	Supporting quotation (therapist number)
Personal access to IBI needed	4 (50) 8	Therapists report that they would like to have access to the IBI to work through it themselves/take a look at it.	*Yes. Uh, (.) it was informative. But I/ at some point I realized that I would've liked to know MORE. (.) Uh then I asked if I could work through the modules myself. This was not possible due to the study design. (T_7)*
Information about patient progress in IBI needed	4 (50) 10	Therapists report that they would like to know about their patients progress in the IBI/what the patients are working on and when.	*But I realized that uh, I would have liked to know EXACTLY what the patient is doing. And this wasn't possible. That was a bit of a shame, but -I could understand why, within the framework of the study. (T_7)*
Therapist material Was not used or used too little	2 (25) 2	Therapists report not having used the therapist material or having used it too little.	*Okay. Well, I have to say, it I/ (smiles) I didn't look at the manual in detail. (T_5)*
Therapist material Too much information	2 (25) 3	Therapists report that the therapist material has too much information or is too long.	*Uh to be honest, I have to say, for me it was (incomp.) uh a good documentation. Uh it was almost too much for me. (T_8)*
Therapist material Nothing missing	1 (12.5) 1	Therapists report that there is no important content missing in the therapist material.	*Uh. (.) Well there's nothing coming to my mind right now/ But as I said, I didn't look at it in detail. But I didn't notice anything in particular where I would've thought that it is missing. Uh or is/ is disturbing in that sense. (.) Yes. (T_5)*
Symptomatology			
Positive influence on symptoms	3 (37.5) 3	Therapists report a positive influence on patient's symptoms.	*But uh it shows that the patient actually is better able to, by applying these emotion regulation skills, uh on one hand can exert more* [...] *control. That is really relevant with regard to the patients' disorder… And uh (.) because yes because of this, their symptoms were uh reduced. Well, they are not completely gone but an improvement is noticeable. And it could be, well I can't/ I can't really say if this is because of the study or the therapy or if it's just a cumu/ cumulative effect. But I can imagine that it also uh happened because of it. (T_4)*
Too early to tell if there is symptom change	2 (25) 2	Therapists report that it is too early to tell if there is a change in patient's symptoms.	*Uh it is difficult for me to tell because I can't estimate at this point if it is uh a stable change or uh just ju/ variance that's showing up in the process. (T_4)*
No symptom changes observed	1 (12.5) 1	Therapists report that no symptom change was observed or that a statement can't be made with regard to symptoms.	*I didn't notice any. It would probably be necessary to ask the patient. (.) (T_6)*
Therapeutic relationship			
No influence on therapeutic relationship	5 (62.5) 5	Therapists report no influence on the therapeutic relationship due to the intervention.	*Therapy/ I don't think that it had an influence on the therapeutic relationship. No. (..) No, really not. (T_7)*
Relationship strengthened due to broader therapy offer	3 (37.5) 4	Therapists report that the therapeutic relationship was strengthened due to a broader therapy offer being made with the intervention.	*Oh, good question. (4) Uh. (.) Well, I think with one patient it did have an influence in that sense that/ that they uh thought that it was very nice that we're making such an effort and offering them various things. And that the patient was more motivated because of this. Or still is. Still is. Uh for therapy and also for the IBI. (T_8)*
Negative influence due to increased workload for patients	2 (25) 2	Therapists report a negative influence on the therapeutic relationship due to an increased workload for the patients.	*Uh (approving) uh I have the feeling that if so, then it would PROBABLY be negative in that sense, because uh (.) I think that the patient wasn't THAT excited about any additional exercises* [...] *(T_3)*

Main theme<br>*Subtheme*	N (%) coded, no. of segments	Definition	Supporting quotation (therapist number)
Negative influence because only available for patients in the outpatient clinic	1 (12.5) 1	Therapists report a negative influence on the therapeutic relationship because the intervention was only available for patients in the outpatient clinic.	*... when I had to tell patients that the online therapy is only available uh (.) for patients in the outpatient clinic. (T₆)*
Less time needed for building of therapeutic relationship	1 (12.5) 1	Therapists report that less time was needed for the building of the therapeutic relationship due to the intervention.	*So. Yes. I would say emotion focused the/ uh, uh interventions were possible earlier on in the therapy session. Earlier than otherwise possible for me, because I probably would have focused more on building the therapeu/ therapeutic relationship and perhaps would have waited a little longer with these types of interventions. Because these are somewhat challenging for some patients and this way I dared to use them a little bit earlier. And I feel like this was positive for the patient too, not negative. In my opinion. (T_5)*
More relationship credit available for interventions in session	1 (12.5) 2	Therapists report more relationship credit available in session and this enabled certain interventions in FTF sessions.	*Uh. (..) Yes. Well, I can imagine that, for me, that it uh. Yes well, if some things were rather new for the patient and the patient already knew some things because of this intervention, that it was also easier for me, well I perhaps started earlier with uh, certain interventions for example. (.) Uh, exactly. (T_5)*
Positive influence on therapeutic relationship since patients have more resources	1 (12.5) 1	Therapists report that patients have resources due to the intervention and this has had a positive influence on the therapeutic relationship.	*And in the process, I mean while it wasn't explicitly a topic for us, I had the impression that because uh emotions were a topic from time to time uh that the patient was already educated on the subject or that they already had some knowledge about it because of this online-program and thereby it was possible to promote resources differently and use them and this again was helpful for the relationship. So, I would say over the course of the sessions it was more beneficial than uh in the beginning, yes, (4) yes. (T_1)*
More contact with patients	1 (12.5) 1	Therapists report that there has been more/a closer contact with the patients due to the intervention.	*Uh (.) well, I have the impression that also in other therapies, which don't use REMOTION, uh that I also talk more about (.) basics about emotions and that I do this time and time again, because I have the impression that some patients need like a reminder from time to time, on why emotions are important, what they are showing us, how we notice them and that in particular this part is (.) uh important but also the aspect of under- and overregulation, I noticed (.) that I myself also gained more awareness about this and (smiles) I look at conversations a bit differently now and uh (.) uh I get into contact with the patients more (.) through these topics. Or also the focusing, that I also use these methods more consciously, because somehow these are small, a/ well, simple methods that can be integrated easily. And I would say that this has (.) uh (.) in general already influenced the process, also in other sessions (.) I noticed. These are by all means things I already learned about or that I already know but that like (.) uh are more present again and I've noticed again that they are relevant in almost all patient relationships and stories, yes. (T_1)*
Blended format			
IBI and FTF not integrated enough	7 (87.5) 11	Therapists report that the IBI is not integrated enough into the FTF therapy.	*Unfortunately, not really because of the low frequency of the therapy sessions. It wasn't really a topic for us because there were so many other things to talk about and it was very difficult to uh include this intervention. (T_3)*
IBI transforms FTF	7 (87.5) 20	Therapists report that the IBI transforms FTF positively.	

Main theme<br>*Subtheme*	N (%) coded, no. of segments	Definition	Supporting quotation (therapist number)
IBI enables new elements in FTF sessions	3 (37.5) 9	New topics, content and processes were enabled in the FTF sessions.	*Yes. Uh, it was really helpful, that / that uh the patient was able to continue working. Uh, uh outside of the therapy session, that they received information uh, that/ uh (.) that we / also theoretical information. And that the patient was able to practice that without it being discussed in therapy. And that they then took these topics and brought them into therapy sessions. I found that REALLY helpful. (T_7)*
IBI allows outsourcing of elements from FTF sessions	4 (50) 7	Therapists report that the IBI allowed the outsourcing of certain elements from FTF sessions/facilitated the work in FTF sessions.	*Uh. Well, it was helpful that I uh, that the patients already received uh a lot of psychoeducation about the nature of emotions. Uh the functionality well like the general things, adaptive, maladaptive and so on. And because of this I didn't need to make such an effort in the sessions. And (.) uh yes it complemented each other very well. Well, this was uh very good and very helpful actually. This has already like paved the way for me and my work or has facilitated it almost. (T_4)*
IBI allows certain topics to be discussed earlier in FTF sessions	3 (37.5) 4	Therapists report that the IBI allowed certain topics to be discussed earlier in FTF sessions.	*I'm I/ I have the feeling that I started earlier with the emotion focused intervention than I usually would. Perhaps with the knowledge that/ that they are dealing with topics like emotions and uh feeling emotions and coping with them, that they are dealing with it more. And maybe, if the patient didn't have the intervention, they would have felt more challenged by, certain interventions. I think this might be the case. That it was possible to do something like this earlier in therapy. Uh. (.) That's what comes to mind right now. (T_5)*
No influence on session structure	6 (75) 7	Therapists report that there has been no influence on the session structure of the FTF therapy.	*Uh no. It hasn't changed. (T_4)*
Patients integrated intervention into FTF	6 (75) 11	Patients integrated elements from the IBI into the FTF sessions.	*Uh. (.) not very much. It has changed insofar as the patient uh (..) has brought in some thoughts about the intervention. (.) Uh, but that didn't change therapy that much. (T_7)*
Enables work independent of time and place for patient/useful in daily life	6 (75) 9	Therapists report that the intervention enables work independent of time and place/is useful in daily life.	*It i/ is generally a good thing. Uh in comparison to the FTF therapy there's the benefit that it is/ the information is always available. The patient can choose for themselves WHEN the patient wants to read which information and do which exercises. Perhaps also in a moment when the topic is relevant. And in the FTF therapy it is determined, what time and place uh, one has to work on it. (T_6)*
Therapists integrated intervention into FTF	4 (50) 7	Therapists integrated elements from the IBI into the FTF sessions.	*Or I have started for example at the beginning of/ of the therapy session uh, yes, also like to put a focus on/ on emotions for a short moment or more generally how the mood is at the moment. [...] And uh at this point the patient told me that uh the/ they know this already a little bit uh from the intervention. Yes, the patient made a connection there in this moment. Yes, like that. But one could definitely integrate more in the future, I think. (T_5)*
Generally a positive experience	4 (50) 8	Therapists wish for more offers of blended treatment or report a generally positive experience with the intervention.	*I wish that there would be more of these combinations in the future. So. Well, I can also imagine that it will be like that in the future. (.) Exactly. (T_5)*

Main theme<br>*Subtheme*	N (%) coded, no. of segments	Definition	Supporting quotation (therapist number)
IBI and FTF complement each other	4 (50) 6	Therapists report that the IBI is an addition to/complements the FTF therapy.	*There I thi/ an online program like this is a good addition, also to work on emotions for example or the coping with emotions, uh, on days where one is not in the sessions. So. Well, I see this quite positively. (T_5)*
Flexible timing of intervention desired	2 (25) 6	Therapists wish that the starting point/timing of the intervention could be chosen flexibly.	*Uh I think uh (thoughtful). (.) I couldn't say what was impeding really. I think it would have been like an addition to have access to it somehow and to look at it and perhaps also the timing would be something, that I have thought of myself. That perhaps/ uh, yes this probably isn't possible because it is important for the study that all patients start at the same time, but that somehow it would be possible to decide on the pace as a therapist. Well, if it wasn't about the study but about the real life setting, then I think it would be extremely important to decide at which moment the app is being used and at which pace the different steps are being worked through. This would be very very helpful (...) (T_3)*
IBI does not replace FTF	1 (12.5) 1	Therapists report that the IBI does not replace FTF therapy.	*Uh it was very concise, well it is really focused ONLY on emotions and I had the impression that it needs something else alongside with it. Well like psychotherapy, so that the clients can/ the patients can benefit from it. Well, (.) uh as an intervention PURELY on its own, I don't know if the patients can (.) yes/ yes can benefit from it (.) exactly. (T_1)*

Note. To further ensure anonymity, he/she was substituted with the patient/they/them in the quotations and the sentence structure was adapted accordingly. Equivalent patient and therapist numberings in the category system do not indicate a psychotherapy dyad (e.g. T_1 was not the therapist of P_1 etc.). The category system was translated into English from German.
